# Using UPLC–MS/MS to Evaluate the Dissemination of Pyriproxyfen by *Aedes* Mosquitoes to Combat Cryptic Larval Habitats after Source Reduction in Kaohsiung in Southern Taiwan

**DOI:** 10.3390/insects11040251

**Published:** 2020-04-16

**Authors:** Ying-An Chen, Yi-Ting Lai, Kuo-Chih Wu, Tsai-Ying Yen, Chia-Yang Chen, Kun-Hsien Tsai

**Affiliations:** 1 Institute of Environmental and Occupational Health Sciences, College of Public Health, National Taiwan University, No. 17, Xuzhou Road, Taipei 100, Taiwan; f03844002@ntu.edu.tw (Y.-A.C.); feynmenfan@gmail.com (T.-Y.Y.); dbms@ntu.edu.tw (C.-Y.C.); 2 Master of Public Health Program, College of Public Health, National Taiwan University, No. 17, Xuzhou Road, Taipei 100, Taiwan; laiyiting0824@gmail.com; 3 National Mosquito-Borne Disease Control Research Center, National Health Research Institutes, No. 211, Zhongzheng 4th Rd., Qianjin Dist., Kaohsiung City 801, Taiwan; wukuochih@gmail.com; 4 Institute of Food Safety and Health, College of Public Health, National Taiwan University, No. 17, Xuzhou Road, Taipei 100, Taiwan; 5 Department of Public Health, College of Public Health, National Taiwan University, No. 17, Xuzhou Road, Taipei 100, Taiwan; 6 Department of Entomology, College of Bio-Resources and Agriculture, National Taiwan University, Insect Building: No. 27, Ln. 113, Sec. 4, Roosevelt Road, Taipei City 106, Taiwan

**Keywords:** pyriproxyfen, autodissemination, integrated vector management, UPLC–MS/MS, bioassay

## Abstract

The policy regarding mosquito control strategies in Taiwan is based on integrated vector management (IVM). The major approach is source reduction via collaboration by both residents and governments. However, small and cryptic habitats of dengue vectors are hard to find and eliminate in urban communities. Therefore, this study evaluated a complementary approach that targeted cryptic habitats by utilizing mosquitoes themselves as vehicles to transfer an insect growth regulator, pyriproxyfen (PPF), to their breeding sites; the amount of PPF in breeding water was determined with ultra-performance liquid chromatography coupled with tandem mass spectrometry (UPLC–MS/MS). A bioassay conducted by introducing ten late-instar larvae into PPF solution was performed to assess emergence inhibition (EI). PPF was found at 0.56 ± 0.04 ng in 25 mL of water by dissemination via ten *Aedes aegypti* mosquitoes exposed to 0.01% PPF, leading to 100% EI. After the community-level source reduction, a field trial in Kaohsiung in Southern Taiwan showed that 30.8–31.5% of cryptic ovitraps reached EI ≥ 50% one month after spraying 0.01% PPF in microhabitats favored by mosquitoes. IVM in parallel with residual spraying of PPF on resting surfaces of mosquitoes could serve as a simple and complementary approach to reduce cryptic larval sources in urban communities in Southern Taiwan.

## 1. Introduction

Taiwan is an island located in East Asia that spans both tropical and subtropical climates (22–25° N and 120–122° E). The most important vector-borne disease in Taiwan is dengue fever (DF), typically occurring in Southern Taiwan, which harbors both major vectors that transmit dengue viruses (DENVs), namely, *Aedes aegypti* and *Aedes albopictus* [1]. *Ae. albopictus* is distributed throughout the island at an elevation of less than 1000 m, while *Ae. aegypti* is distributed in only Southern Taiwan below the Tropic of Cancer (23.5° N) and favors urban areas, which enhances the spread of dengue [2]. DF outbreaks have mostly occurred in Kaohsiung city, Tainan city, and Pingtung County in Southern Taiwan in the rainy season from June to December [3]. Dengue outbreaks in Taiwan have usually started from imported cases from Southeast Asia and then developed into indigenous outbreaks [4]. To control DF and minimize indigenous outbreaks, integrated vector management (IVM) is implemented in Taiwan following the recommendations of the Centers of Diseases Control, Taiwan (Taiwan CDC), and World Health Organization (WHO) [5,6,7]. Nonchemical and chemical vector control methods are integrated with other disease control methods, such as surveillance, diagnosis, and treatment, at national and subnational levels. In Taiwan, primary prevention measures emphasize source reduction via collaboration among residents, communities, and governments. To avoid the development of severe insecticide resistance, chemical pesticide spraying is conducted only during indigenous DF outbreaks to kill DENV-carrying *Aedes* mosquitoes. The secondary and tertiary prevention measures are to enhance disease surveillance and to control the mortality rate by improving health care quality. Under these prevention measures, the annual DF incidence in Taiwan was controlled to less than 10 cases per 100,000 individuals for two decades, except for a recent outbreak in 2014 and 2015, with a total of 15,492 indigenous cases (61.3 cases per 100,000 individuals) in 2014 and 43,419 cases (168.6 cases per 100,000 individuals) in 2015 reported by the Taiwan CDC [8]. The DF outbreaks in 2014 and 2015 prompted the use of novel vector control methods to complement flaws in the current strategies. One major difficulty in targeting *Aedes* vectors is the multiplicity of larval habitats, mostly represented by cryptic larval sources that are hard to find and eliminate by routine source reduction [9].

To tackle the cryptic breeding sites of *Aedes* mosquitoes, an approach called “autodissemination” was developed by utilizing adult mosquitoes themselves as carriers to transfer insecticide to larval sources through their resting and oviposition behaviors [9,10,11]. This approach has two requirements. First, a tiny dose of insecticide delivered to larval sources must have a strong effect by reducing the levels of immature mosquitoes but a relatively weak effect on the carrier mosquitoes. Pyriproxyfen (PPF), as an insect juvenile hormone analog that primarily affects larval–adult transformation, is an ideal insecticide [12,13]. The minimum EI_50_ (50% inhibition of adult emergence) values against *Ae. aegypti* and *Ae. albopictus* larvae were estimated to be only 0.008 and 0.016 ppb, respectively, in the laboratory susceptible strain [14]. In contrast, PPF has neither lethal nor repellent effects on adult mosquitoes [11,13]. Second, insecticides can be picked up by adult mosquitoes during their stay on PPF-treated surfaces and then transferred to nearby breeding sites. Previous studies created PPF-dusted dissemination stations and found, through scanning electron microscopy or by using a fluorescent powder, that PPF can adhere to the legs and bodies of mosquitoes [11,15,16]. 

In this study, laboratory test and field trial were conducted in Kaohsiung city, where 59% (34,722/58,911) of the DF cases were distributed during the outbreak from 2014 to 2015 [8]. A new form of dissemination sites using PPF residual spray on the resting surfaces favored by *Aedes* mosquitoes was evaluated to determine the efficiency of emergence inhibition at cryptic sources in the field trial. Results from field trial showed that residual spraying of PPF in the microhabitats favored by mosquitoes had a modest effect on reducing cryptic larval sources in urban communities of Southern Taiwan. Moreover, this study was able to observe trace doses of PPF being disseminated into breeding water using ultra-performance liquid chromatography coupled with tandem mass spectrometry (UPLC–MS/MS). The UPLC–MS/MS-based analytical method developed in this study successfully detected PPF at concentrations below the reported EI_50_ for *Ae. aegypti* and *Ae. albopictus* and became a validated method for detecting PPF in the water.

## 2. Materials and Methods

### 2.1. Laboratory Test of PPF Being Carried and Disseminated by Adult Mosquitoes

**Figure 1:**
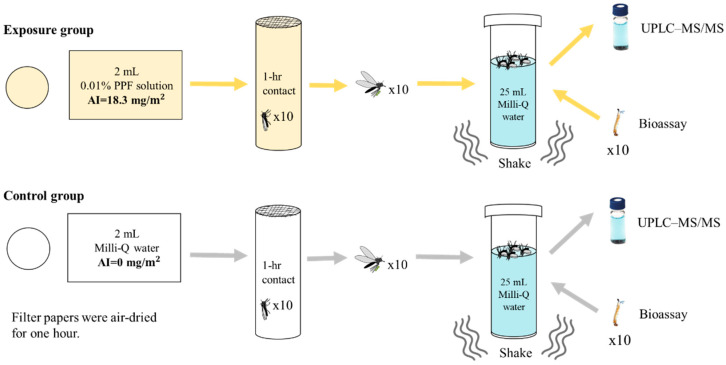
Experimental diagram of mosquito exposure to pyriproxyfen (PPF) and dissemination to a small water container in the laboratory. Ten mosquitoes were trapped in the cylinder tube encircled by the treated filter papers for one hour. Mosquitoes were then transferred and shaken with 25 mL of Milli-Q water for three minutes. Five milliliters of water was removed for ultra-performance liquid chromatography coupled with tandem mass spectrometry (UPLC–MS/MS) analysis, and the remaining 20 mL of water was used for the bioassay by introducing ten late-instar larvae to calculate emergence inhibition (EI).

Two milliliters of 0.01% w/w PPF solution (manufactured by Chung Hsi Chemical Plant, Taipei, Taiwan) was evenly sprayed on 3M filter paper as the exposure group. The control filter paper was sprayed with 2 mL of Milli-Q water. The active ingredient (AI) of PPF on the filter paper of the exposure group was 18.3 mg/m^2^. The filter papers were ventilated for one hour to dry and then used to encircle the inner surface of the plastic cylinder tube. Ten laboratory-reared female adult *Ae. aegypti* mosquitoes were kept in the tube in contact with the filter paper for one hour and transferred to another tube containing 25 mL of Milli-Q water. Ten exposed mosquitoes were shaken gently with 25 mL of Milli-Q water for three minutes to simulate the maximum dose of PPF per mosquito to disseminate into a small amount of water. Five milliliters of the above water sample was taken for quantitation of PPF using UPLC–MS/MS. The remaining 20 mL water sample was used for the mosquito bioassay. The exposure experiment was repeated three times. The experimental diagram is shown in Figure 1.

Additionally, to monitor whether PPF particles on filter paper can adhere to mosquitoes, luminous paints and powders (BioQuip Products) were mixed with 0.01% PPF solution and applied to filter paper in a preliminary test. Ten mosquitoes were brought in contact with the treated filter paper for one hour, and then the mosquitoes were frozen in a −20 °C freezer for 5 min to halt their activity. Contamination with the fluorescent powders on mosquitoes was checked under a 365 nm UV LED (Everlight Electronics, Co., Ltd. Taiwan) and photographed by the microscope (Leica Microsystems, Wetzlar, Germany).

### 2.2. Field Trial in Kaohsiung City

**Figure 2:**
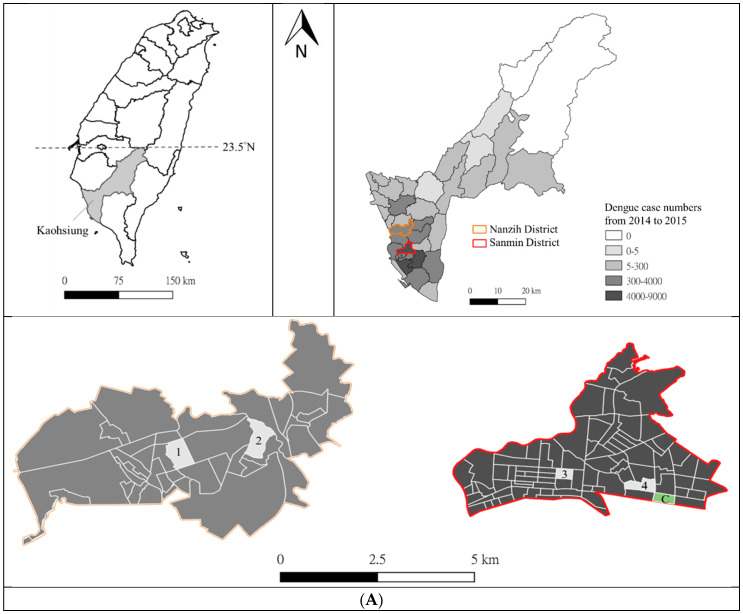

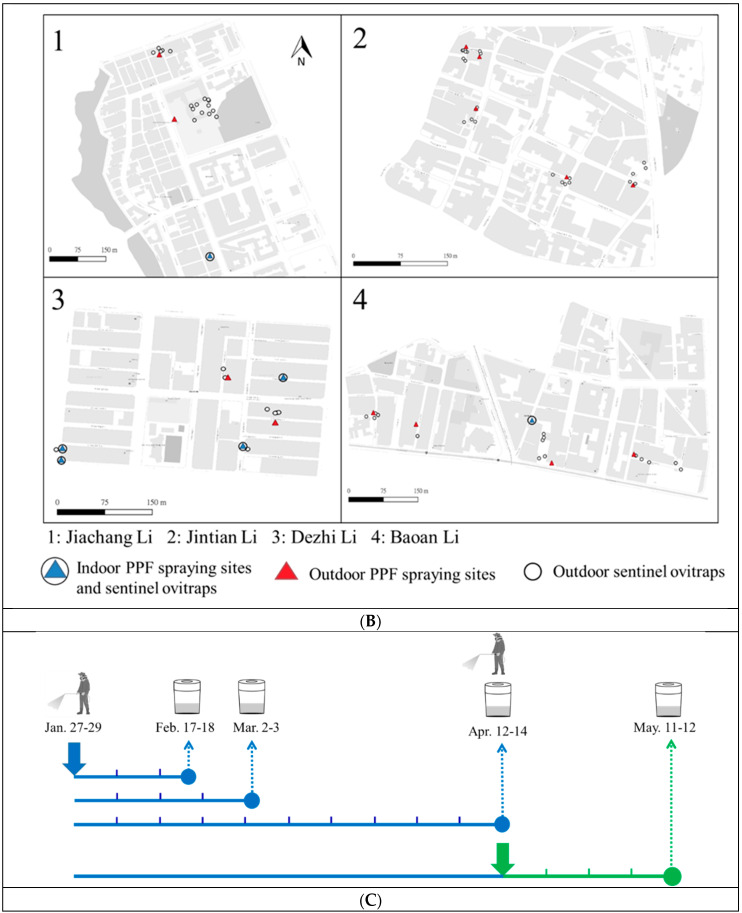
Map of the PPF field trial. (**A**) shows the location of Kaohsiung city in Southern Taiwan and the distribution of dengue fever (DF) cases in the districts of Kaohsiung. Two districts in Kaohsiung, namely Nanzih and Sanmin, were selected for the field trial. PPF field trial was conducted in four Li (block 1: Jiachiang Li; block 2: Jintian Li; block 3: Dezhi Li; block 4: Baoan Li) and one control Li, Baoshi Li (block C). (**B**) shows the distribution of indoor and outdoor PPF spraying sites and sentinel ovitraps. There were 6 indoor basements subjected to PPF spraying, and a total of 30 sentinel ovitraps were placed at the corners of the basements (circled blue triangles). A total of 67 outdoor sentinel ovitraps (dots) were placed within 10 m of the nearest spraying microhabitats (red triangles represent the center of the outdoor spraying microhabitats). (**C**) shows the timeline of PPF field trial. Each interval on the line represents the time of one week. The blue and green down arrows indicate the first and second spraying timepoints. The circles with the up arrows indicate the time points of water collection from sentinel ovitraps (total four times).

A total of 19 PPF spraying sites and 97 sentinel ovitraps were set in four “Li” (the administrative name for “urban village” in Taiwan), namely, Dezhi and Baoan Li in Sanmin District, and Jiachang and Jintian Li in Nanzih District (Figure 2A,B). The field trial was conducted after the 2014–2015 outbreak, and testing was conducted after source reduction at the DF prevention stage, from January to May 2016. Sanmin District had the most dengue cases in Kaohsiung, with 8671 (25%, 8671/34,722) cases reported during the 2014–2015 outbreak, while Nanzih District had 965 (3%, 965/34,722) cases reported. There were three control sentinel ovitraps placed in the nearby Baoshi Li in Sanmin District without PPF spraying. PPF solution with a concentration of 0.01% w/w (manufactured by Chung Hsi Chemical Plant, Taipei, Taiwan) was sprayed at indoor and outdoor microhabitats favored by mosquitoes using a power sprayer (manufactured by Chaang Cherng Co., Ltd., Kaohsiung, Taiwan). The flow rate of the sprayer was 1.0–1.2 L/min, and each square meter was evenly sprayed for 2–3 s. The spraying microhabitats included indoor basement walls, outdoor sewer ditches, flowerpots, canvas carports, nonflowering plants, and other artificial settings. The above microhabitats had been recorded to have *Aedes* mosquitoes. The sentinel ovitrap was a 1.2 L black plastic bucket with a hole (4 cm diameter) on the top. A 250 mL beaker filled with 75 mL of water was placed inside the bucket. Sentinel ovitraps were placed indoors at the corners of basements and outdoors at sheltered and cryptic sites within 10 m of the nearest spraying microhabitats. The ovitraps were placed post-spraying to avoid direct contamination by the PPF spray and to evaluate the dissemination effect only.

PPF spraying was conducted twice: 27–28 January and 12–13 April. To evaluate the PPF dissemination efficacy at different time points, water samples from sentinel ovitraps were collected at intervals of 2–3 weeks (19 days post-spraying, first collection), one month (33 days post-spraying, second collection), and over two months (74 days post-spraying, third collection) after the first spraying in both districts in January. For the second spraying in April, 50 water samples were collected in Sanmin District after one month (27 days post-spraying, fourth collection) (Figure 2C). Mosquito eggs and larvae and dead bodies of *Aedes*, *Culex*, and *Armigeres* species and other creatures in the sentinel ovitraps were checked and removed before the next sampling. Twenty milliliters of a water sample from each sentinel ovitrap was taken for the bioassay, and five milliliters of water was stored in a 4 °C refrigerator for further analysis using UPLC–MS/MS. The water volume was kept at 75 mL in the sentinel ovitraps after each collection.

The additional residual test was performed one month after the first spraying by wetting the filter paper on the spraying surfaces and reimmersing the filter paper into Milli-Q water to perform the bioassay. The residual test was conducted at 17 spraying sites (two sewer ditch sites were excluded). The EI% of the water soaked with the filter paper was recorded, and the proportion that reached ≥ 50% EI was calculated.

### 2.3. Mosquito Bioassay

Ten mosquito larvae (3rd to 4th instars) of *Ae. aegypti* were introduced into a 20 mL tested water sample. Water without contamination of PPF was used as controls. Yeast powder (2.5 mg) was added as a nutrient for the larvae every two days. The test was terminated when all the late-instar larvae in the controls emerged into adults (usually five days in our laboratory test). The adult EI% was calculated as 100 − (T * 100/C), where T indicates the percentage of successful emergence in the treated group, and C indicates that in the control group [17]. For the laboratory tests, Fisher’s exact test was performed to compare the difference in EI between the exposure and control groups. For the field trial, the proportion of collected water samples from sentinel ovitraps reaching EI ≥ 50% (considered effective PPF activity in our study) was calculated per Li at four collection time points. Kruskal–Wallis and Dunn’s multiple comparisons were conducted to compare the EI differences among Li, indoor/outdoor microhabitats, and collection time points. All statistical analyses were performed by R version 3.5.1.

### 2.4. Detection and Quantitation of PPF in Water Samples Using UPLC–MS/MS

The 5 mL water sample used for the instrumental analysis was premixed with 1.25-mL acetonitrile (ACN), and 1.25 mL of the mixture was filtered with a polytetrafluoroethylene (PTFE) syringe filter (0.20 μm; Macherey-Nagel GmbH & Co. KG, Düren, Germany) into a vial. The analytical standard pyriproxyfen (purity 98.9%, 100 μg/mL in ACN; AccuStandard Inc., New Haven, CT, USA) was diluted with Milli-Q water at the same ratio as the water samples (water: ACN = 4:1). To construct a standard curve, standard solutions were prepared by 5-fold serial dilution to obtain the following concentrations: 10,000, 2000, 400, 80, 16, and 3.2 ng/L. All solutions were prepared one day before the analysis and were stored at 4 °C. Chromatographic gradient separation was performed on a Waters ACQUITY UPLC system (Waters, Milford, CT, USA) with a Waters BEH C_18_ column (50 × 2.1 mm, 1.7 μm) at 55 °C. The mobile phase was composed of (A) 5 mM ammonium acetate_(aq)_ and (B) methanol (LC grade) and the flow rate was 0.6 mL/min. The gradient started with 50% B for 0.5 min, increasing to 90% B in 2 min, holding for 0.3 min, and returning to 50% B for re-equilibration in 1.7 min; the total run time was 4.5 min. The injection volume was 40 μL, and each sample underwent 3 replicate injections followed by a blank (methanol) injection for washing.

The UPLC system was coupled with a Waters Quattro Premier XE triple-quadrupole mass spectrometer at multiple-reaction monitoring (MRM) mode with positive electrospray ionization (ESI+). The ion source temperature was 120 °C, and the cone gas flow rate was 100 L/h. Desolvation gas was nitrogen with a flow rate of 900 L/h at 450 °C. The capillary and cone voltages were set at 3.0 kV and 25 V, respectively. Two ion transitions of m/z 322.1 → 95.7 at 15 eV collision energy and m/z 322.1 → 184.9 at 25 eV were monitored for quantitation and confirmation of PPF, respectively, and the dwell time was 0.1 sec at each ion transition. The limit of detection (LOD) and limit of quantitation (LOQ) were determined by signal-to-noise ratios (S/N) of 3 and 10, respectively.

## 3. Results

### 3.1. Laboratory Simulation Test of PPF Being Carried and Disseminated into Small Water Containers

PPF exposure and dissemination experiments were conducted on *Ae. aegypti* in the laboratory (Figure 1), and the EI% from the mosquito bioassay and the quantity of PPF in the tested water are shown in Table 1. The EI% of the exposure groups was 100%. In contrast, in the control groups, all larvae became adult mosquitoes (EI = 0%), and no PPF was detected. Fisher’s exact test showed that the EI was significantly different between the exposure and control groups (*p* < 0.001).

**Figure 3:**
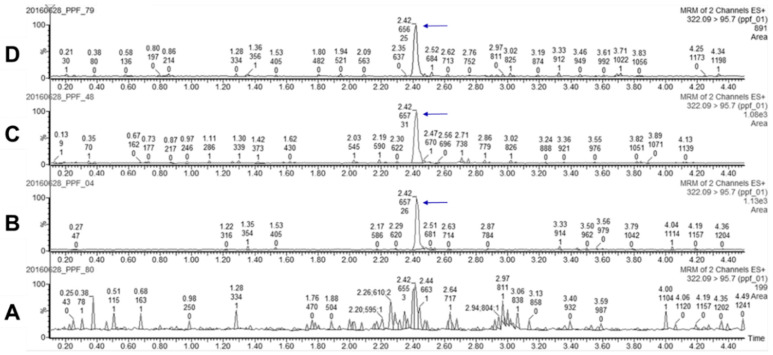
UPLC–MS/MS multiple-reaction monitoring (MRM) chromatograms of (**A**) PPF-untreated control, (**B**) 16 ng/L of PPF standard, (**C**) laboratory water sample from PPF exposure group, and (**D**) field water sample with EI 100%. The PPF peak appeared at 2.42 min (arrow, retention time) with response of ≈1000 in the PPF standard (16 ng/L) and in lab and field water samples with EI 100%.

The retention time of the PPF at m/z 322.1 → 95.7 was 2.42 min (Figure 3). The LOD and LOQ of PPF concentrations in water samples were 0.52 ± 0.24 ng/L and 1.73 ± 0.78 ng/L, respectively. Quantitation of PPF in water samples from the laboratory exposure groups showed that each mosquito could disseminate 0.04–0.08 ng (average = 0.056 ng) of PPF into 25 mL of water after exposure to 0.01% PPF-treated filter paper (Table 1). The average PPF concentration in water samples from the exposure groups was 22.47 ng/L (ranging from 16.1 to 33.6 ng/L), leading to 100% EI of *Ae. aegypti* in the laboratory.

**Figure 4:**
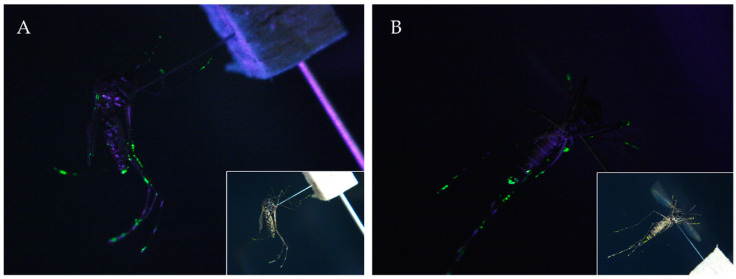
Mosquito of *Ae. aegypti* showed contamination as fluorescence under UVA light (365 nm), showing that mosquitoes carry and disseminate PPF primarily through abdominal and tarsal contact. (**A**) lateral view; (**B**) abdominal view.

The additional fluorescence test showed the mixture on the filter paper (PPF solution mixed with fluorescent powder) adhered primarily to legs of the mosquitoes (Figure 4). Other body parts of the mosquitoes showed relatively little contamination in terms of visible fluorescence.

### 3.2. PPF Dissemination Efficacy in a Field Trial in Kaohsiung

**Figure 5:**
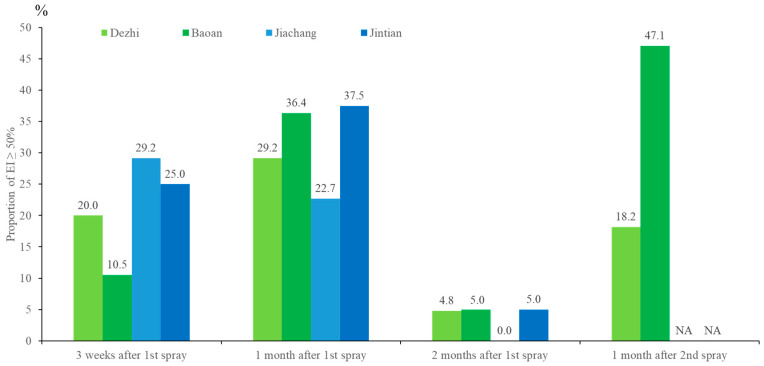
Proportion of sentinel ovitraps reaching EI ≥ 50% in the field. Sentinel ovitraps in Jiachiang and Jintian Li from Nanzih District were not monitored in the fourth collection (NA = not applicable).

There were 97, 95, 86, and 39 water samples collected from the sentinel ovitraps from the first to fourth collection, respectively. The overall proportions of sentinel ovitraps containing mosquito eggs, mosquito larvae, dead mosquito adults, and dead bodies of other creatures (including moth fly, fruit fly, nonbiting midge, ant, spider, weevil, and snail) were 10.7% (34/317), 2.8% (9/317), 0.9% (3/317), and 38.8% (123/317), respectively. There was no EI or PPF detected from the three control sentinel ovitraps in Baoshi Li throughout the field trial. The proportion of sentinel ovitraps reaching EI ≥ 50% in the four Li at four collection time points is shown in Figure 5. There were no significant spatial differences in EI% among districts (χ^2^ = 0.15, d.f. = 1, *p* = 0.70), Li (χ^2^ = 0.25, d.f. = 3, *p* = 0.97), and indoor/outdoor microhabitats (χ^2^ = 1.53, d.f. = 6, *p* = 0.96), but significant temporal differences (χ^2^ = 54.80, d.f. = 3, *p* < 0.001) were observed among the four collection time points. Within one month post-spraying, an average of 27.6% (range = 10.5%–47.1%) of the sentinel ovitraps reached EI ≥ 50%. This effect significantly decreased two months after spraying, at which the proportion with EI ≥ 50% was only 0%–5% in the third collection. 

**Figure 6:**
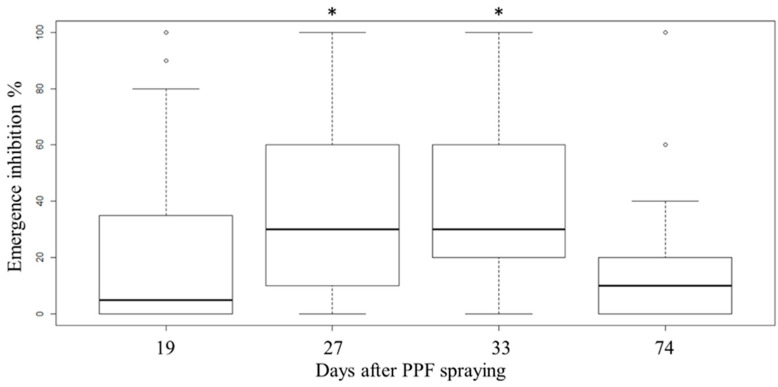
Box plot of EI% in sentinel ovitraps at 19, 27, 33, and 74 days after PPF spraying. Multiple comparison of EI% showed that after one month (27 and 33 days, median = 30%) the value was significantly higher (adjusted *p*-value ≤ 0.002, labelled with *) than those after three weeks (19 days, median = 5%) and after two months (74 days, median = 10%) by the Bonferroni correction for post-hoc Dunn’s pairwise tests.

The EI% of post-one-month (27–33 days, median = 30%) was significantly higher than that of post-three-weeks (19 days, median = 5%) and post-two-months (74 days, median = 10%) (Figure 6). The PPF dissemination effect mainly appeared one month after spraying, which could lead to half of the sentinel ovitraps reaching 10%–60% EI (median = 30%, mean = 37%) and 30.8%–31.5% of ovitraps reaching EI ≥ 50%. In contrast, sentinel ovitraps placed for 2–3 weeks and over 2 months showed less effect on PPF dissemination to inhibit the emergence of adult mosquitoes in the field. The residual test performed one month post-spraying showed that 47.1% (8/17) of dissemination sites (direct contact) retained the efficacy of causing EI ≥ 50% in late-instar larvae.

In addition, 21 field water samples (EI ranging from 10% to 100%) with low levels of organic matter were chosen for PPF detection by UPLC–MS/MS. Five field water samples that had 70–100% EI showed positive PPF signals (Figure 3D), and the PPF concentration in these samples was estimated to be 8.6–11.0 ng/L using external standard calibration.

## 4. Discussion

This study evaluated PPF dissemination efficacy in both laboratory and small-scale field trial in communities. Compared to previous studies, there were three novel aspects to the design. First, previous studies measured outcomes solely by observing EI in immature mosquitoes but lacked direct evidence of the existence of PPF in breeding water. Only a few studies measured the attachment and retention of PPF on *Aedes* mosquitoes [18], and the PPF residual in the water from sentinel cups was measured using LC–MS/MS [19,20]. This study developed a UPLC–MS/MS analytical method for detecting and quantifying PPF in breeding water and further explored the dose–response effect on immature *Ae. aegypti*. To simplify the treatment of water samples for chemical analysis, in this study, we added ¼ volume of acetonitrile to the water samples, which enhanced approximately 25 times PPF signal intensities than those of the original water samples. The limit of quantitation was estimated to be 1.7 ng/L, which was lower than the reported minimum EI_50_ value for *Aedes* mosquitoes (12 ng/L) [13,14]. PPF was detected in the water samples collected from both the laboratory and field at concentrations ranging from 8.6–33.6 ng/L, which resulted in more than 70% EI in *Ae. aegypti* in our study. This result was similar to those of Unlu et al. [19] and Chandel et al. [20], in which the PPF residual in water samples taken from sentinel cups ranged from 4 to 11 ng/L, causing more than 70% pupal mortality in *Ae. albopictus*.

Second, previous studies created a variety of point-source PPF dissemination stations (PPF-treated ovitraps or devices [18,19,21], nets [22,23], tire piles [24,25]) or used an area-wide ultra-low-volume (ULV) spray [24,26]. Only a recent study used a PPF residual spray on small surface areas in animal shelters to successfully reduce *Anopheles* and *Culex* mosquitoes that fed on livestock [27]. Our study evaluated whether resting surfaces of *Aedes* mosquitoes sprayed with PPF solution could become dissemination stations. The simulation test in the laboratory showed positive detection of PPF after mosquitoes were exposed to PPF-treated filter paper. In the field trial, we then sprayed PPF on the resting surfaces favored by *Aedes* mosquitoes after source reduction, such as basement walls, sidewalls of sewer ditches, flowerpots, backs of leaves (nonflowering plants), and surfaces with a dark color, to prepare the dissemination sites. The EI effect was also observed in ~30% of sentinel ovitraps that reached EI ≥ 50%. This study defined EI ≥ 50% as being efficient with a tiny dose of PPF introduced into the water since, under normal rearing conditions, it is rarely possible to induce mortality in more than half the population. Residual spraying is a feasible method and more doable than decorating many point-source dissemination containers. In addition, residual spraying of PPF in a specific localized area may have a better autodissemination effect than ULV spraying, which may have a dilution effect in a wide area [24]. 

Third, this study used commercial, solution-based PPF at a low concentration (0.01%) for application. Previous studies used a granular formulation of PPF (Sumilarv 0.5G), and pretreatment was often required, such as pulverization and grinding into powder [11,13,15] or agitation in organic solvents [22,28,29]. The ULV spray commonly used the 10% PPF concentrate (NyGuard^®^ IGR Concentrate) for application [24,26]. In our study, the diluted 0.01% PPF commercial product could be directly applied for residual spraying without any pretreatment. On the other hand, the concentration of PPF product used in previous autodissemination studies varied from 0.5–10% and could result in larval/pupal mortality of the dengue vector of more than 60% [9,11,21,24,30]. The concentration of PPF product used in our study (0.01%) was relatively low compared to that in other studies, and the result showed that the mean EI% was 37% one month post-spraying. This EI% observed in our field trial was similar to the value from a study by Chandel et al., in which the mean percentage of pupal mortality was 29.7% among cryptic sentinel cups [20]. Although the low concentration of PPF used in the field may have a weak EI effect, it would rarely harm untargeted creatures, making it an ecofriendly strategy to control mosquitoes. During the period of the field trial (27 January to 12 May 2016), the locations that we sprayed PPF did not have any DF cases; therefore, there was no other chemical spraying that may have interfered with our results. The field trial was conducted after source reduction in the communities to target the cryptic sources that were not eliminated. Residents were alert and sensitive when they saw the artificial containers, and some ovitraps were removed, as expected, during this field trial. This is why, in this study, we failed to collect water samples from some sentinel ovitraps that were turned over, nearly dry, or not found. Despite the loss, 83–100% of the water samples were collected, and the organisms inside the ovitraps were recorded on-site. These records showed that the ovitraps were visited by not only mosquitoes but also other creatures, such as moth flies, fruit flies, nonbiting midges, ants, spiders, weevils, and snails, which could also visit and possibly disseminate PPF into the ovitraps from the spraying environment. This could possibly complement the insufficient autodissemination events under low mosquito density after source reduction, which was also a limitation of our study. This study was not continued after May because DF epidemics in Southern Taiwan appeared in June, and intensive vector control (including chemical spraying in the communities for environmental sterilization) was conducted, which could interfere with the study results. By using the residual spraying method, we suggested that not only could PPF be autodisseminated by mosquitoes themselves, but there is also a high possibility that other moving insects in the surroundings visited our spraying sites and disseminated PPF to the cryptic habitats. 

Spatial-temporal heterogeneity could affect the EI effect in autodissemination studies [16,31]. Suman et al. evaluated the spatial-temporal efficacy of PPF autodissemination via dual-treatment autodissemination stations (container type) in the field, and the results showed that efficacy could be observed for 8–12 weeks (2–3 months), and PPF could be detected in sentinel cups up to 200 m from stations [32]. Doud et al. determined the efficacy of truck-mounted ULV applications, and PPF could be detected 23 m from the spray line at a concentration of 1.40 ng/mL and EI of ≥ 82% from the field immediately after spray [26]. Our study showed that the residual spraying method could maintain efficacy for one month within a distance of 10 m from the spraying sites. A recent study evaluating autodissemination efficacy in the control of *Ae. aegypti* on Madeira Island, Portugal, showed that the impact of PPF was cumulative over time both locally and with gradual spatial expansion, presumably due to cumulative PPF dissemination events [16]. Under PPF autodissemination treatment, the juvenile mortality during short (9–11 days) and long (13–29 days) periods was 17.3–23.1% and 21.6–38.4%, respectively, in the aforementioned study [16]. This modest cumulative effect was similar to our result, in which the median and average EI (juvenile mortality) at 27–33 days were approximately 30–40%. Under the urban landscape, there was a large variation in EI (ranging from 0–100%) among all the sentinel breeding sites observed in our study and the study by Seixas et al. [16]. According to the chemical fact sheet reported by WHO, the estimated photolytic half-life of PPF is 17.5–21 days in water and 6.4–36 days in soil under aerobic conditions [33]. The additional residual test in our study showed that almost half of the microhabitats retained PPF one month post-spraying, which could lead to EI ≥ 50% through direct contact. Therefore, this study suggested that by using the residual spraying method to disseminate PPF, the efficacy could persist for one month under the low concentration applied in the urban communities and the environmental fate of PPF exposed to natural variation [34].

DF is not endemic in Taiwan, although it is highly severe in Southeast Asian or South American countries [35,36]. After the 2014–2015 outbreak, DF was controlled to an annual incidence of less than 3.1 per 100,000 individuals owing to the awareness of prompt vector control intervention and case management methods. Once a dengue case is confirmed, the local health and environmental agency initiates a program of insecticide spraying within a radius of 50 m from the case residence and perform comprehensive source reduction [37]. In Taiwan, PPF is applied directly to breeding sites to inhibit the potential growth of mosquito larvae. However, PPF is not as popular as other larvicides, such as chlorpyrifos and *Bacillus thuringiensis israelensis,* which can kill larvae in a relatively short period. In this study, we demonstrated that PPF spraying is a useful and ecofriendly method that can be used in the microhabitats favored by mosquitoes, allowing dissemination by mosquitoes to eliminate the neglected cryptic sites after source reduction. Resistance to PPF is rare in *Aedes* mosquitoes; however, recent studies have discovered reduced susceptibility or cross-resistance to PPF in some *Ae. albopictus* and *Ae. aegypti* populations [38,39,40]. Overall, this study demonstrated the feasibility of using the PPF dissemination strategy as a complementary method to reduce cryptic sources of dengue vectors after source reduction in communities of Southern Taiwan, with the benefits of easy application, low ecotoxicity, and lack of resistance. Follow-ups and comprehensive long-term assessments should be carried out in the future to optimize the implementation and efficacy.

## 5. Conclusions

To our knowledge, this is the first field study to evaluate a PPF dissemination strategy to control *Aedes* mosquitoes in Taiwan. The simulation test conducted in the laboratory showed successful contact and dissemination effects by UPLC–MS/MS analysis, fluorescence testing, and adult EI observation from the mosquito bioassay. Furthermore, a small-scale field trial was also conducted in the dengue hot spot in Kaohsiung. The results showed that PPF residual spraying in microhabitats favored by mosquitoes could become a simple and complementary approach to reduce cryptic sources by 30.8–31.5% in the communities of Southern Taiwan after source reduction. PPF dissemination could maintain the efficacy for one month within a distance of 10 m from the spraying sites. This method could be integrated with the existing vector control strategies to boost efficiency, especially when tackling small and cryptic breeding sites.

## Figures and Tables

**Table 1:** Laboratory simulation tests of PPF being carried and disseminated by *Ae. aegypti*.

Group	Emergence Inhibition of *Ae. aegypti* (%)	PPF Concentration in Water (ng/L) *	Average PPF (ng) Per Mosquito Disseminated into 25 mL of Water †
Control (Water)	0	ND	ND
PPF exposure group 1	100	17.71 ± 0.38	0.044 ± 0.001
PPF exposure group 2	100	33.63 ± 2.63	0.084 ± 0.007
PPF exposure group 3	100	16.08 ± 1.45	0.040 ± 0.004
Average in exposure groups	100	22.47 ± 1.49	0.056 ± 0.004

Values are denoted as the mean ± standard deviation. ND = Not Detected. * External standard calibration was used for calculating the PPF quantity in the water samples. † The values were calculated by the following formula: PPF concentration in water (ng/L) * 0.025 (water volume, L)/10 (mosquitoes).
